# Most Scandinavians Are Born During Summer Time and Less Norwegians Are Born the First Quarter of the Year: A Study Comparing Scandinavian Birth Patterns 2000-2012

**DOI:** 10.5539/gjhs.v6n4p163

**Published:** 2014-04-14

**Authors:** Jan Norum, Anca Heyd, Tove Elisabeth Svee

**Affiliations:** 1 Institute of Clinical Medicine, Faculty of Health Science, University of Tromsø - The Arctic University of Norway, Tromsø, Norway; 2 Department of Radiology, University Hospital of North Norway, Tromsø, Norway; 3 Northern Norway Regional Health Authority trust, Bodø, Norway

**Keywords:** obstetrics, Norway, Sweden, Denmark, kindergarten

## Abstract

**Objectives:**

Summer time is a challenging period in obstetric care as many health care workers are on holiday. We aimed to explore the Scandinavian birth patterns and the Norwegian kindergarten reform’s possible influence on time of delivery in Norway.

**Methods:**

A retrospective analysis using data (2000-12) from the medical birth registries of Denmark, Norway and Sweden was carried out. Annual data for each country were compared. The first five years (2000-2004) period was compared with the periods 2005-2009 and 2010-2012 to clarify any changing trend in month and seasons of delivery. Furthermore, the time period following the Norwegian kindergarten reform (2010-12) was compared with the time period 2000-2009. In total, there were 760,168 Norwegians, 827,354 Danes and 1,354,177 Swedes born during study period.

**Results:**

Whereas the number of deliveries increased in Sweden (24%) and Norway (3%), there was a 12% reduction in Denmark during study period. Comparing seasons, most births (35.3%) occurred during summer time (May-August). In Norway, there was a significant change during study period with fewer children born between January and April (P < 0.04) and more during summer time (P < 0.01). The lower percentage of births during the last quarter of the year was stable in all countries.

**Conclusion:**

Most Scandinavians were born during summer time. During study period a significant shift of births from spring to summer time was observed in Norwegians. So far, the Norwegian kindergarten reform has not influenced on the birth rate between September and December.

## 1. Introduction

Quality of care has been constantly focused in maternity care and Norwegian women have been selected to institution/unit of delivery according to risk factors (Nesheim et al., 2010; Holt et al., 2001). Recently a new national Norwegian reform (Ministry of Health and Care Services, 2009) introduced new guidelines concerning manning of delivery units. Consequently, the four regional health authority (RHA) trusts had to increase the number of obstetricians and midwives to meet the new recommendations for manning. Summer time is a challenging period in obstetric care as many health care workers are on holiday. In a situation with raising health care costs and steadily improving rights of health care workers, strategies to improve cost-effectiveness and annual operating plans are crucial. Pattern of birth is among the necessary data for good planning.

In 2009, a political reform concerning kindergarten services was launched in Norway. Whereas children born before the 1^st^ of September were guaranteed access to kindergarten the following year, those born later did not get similar rights. The date of September 1^st^ was set simply because those who reached school age at this time leave kindergarten and consequently new uptakes can be performed. Since the reform was introduced, some midwives have indicated an increasing number of births during summer time, possibly due to family planning. In this study we aimed to explore birth patterns before and after the introduction of the Norwegian reform and compare these findings with data from our neighboring countries, Sweden and Denmark.

## 2. Methods

### 2.1 Study Design/Settings and Material

In September 2013, we accessed retrospectively the national medical birth registries of the Scandinavian countries Denmark, Norway and Sweden. The aims of the registries are epidemiological surveillance of birth defects and other perinatal health problems in order to detect, as soon as possible, any future increase in rates. The Danish Medical Birth Registry (DMBR) was established in 1968 and has been computerized since 1973. The Medical Birth Registry of Norway (MBRN) contains all births in Norway since 1967. The corresponding Swedish Medical Birth Registry (SMBR) was founded in 1973 and celebrates this year its 40th aniversary.

**Figure 1:**
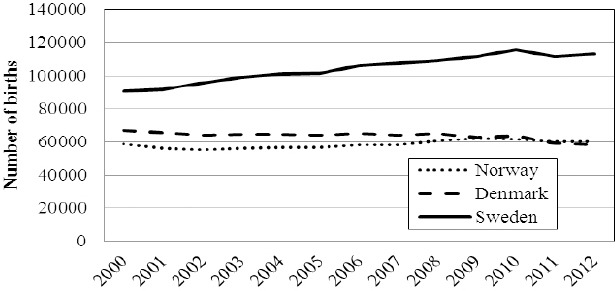
The figure illustrates the annual number of births in the Scandinavian countries during the time period 2000-2012

In this study, the thirteen years time period 2000-2012 was focused. Denmark, Norway and Sweden had, as of July 2012, a population of 5.5 million, 5.0 million and 9.1 million inhabitants, respectively. The corresponding mean annual numbers of births during study period were 63,643 births (Denmark), 58,474 births (Norway) and 104,167 births (Sweden), respectively. The numbers are illustrated in Figure 1. In total, there were 760,168 Norwegians, 827,354 Danes and 1,354,177 Swedes born during study period.

We aimed to clarify any change in birth pattern in Norway and compare any findings with those of our neighboring Scandinavian countries. The following data were implemented in our database: Number of births per month and year in each country. We hypothesized an increasing share of births occurring during summer time and fewer births taking place between September and December, especially after the year of 2009. The hypothesis was based on suggestions/indications from midwives following the implementation of the Norwegian kindergarten reform in 2009 (Ministry of Education and Research, 2008). There were also internal reports of increased pressure on the obstetric health care service during summer time. Furthermore, we noticed Norwegian newspapers indicating couples aiming for delivery before September 1^st^ and consequently obtaining a guaranteed uptake of their children in the kindergarten the following year (Ministry of Education and Research, 2008).

### 2.2 Quality Control, Statistical Analysis and Authorisation

Anonymous and aggregated data were recorded retrospectively from the medical birth registries in Denmark, Norway and Sweden. The aggregated data were imported into a database at the NNRHA trust and analyzed. The Microsoft Excel 2007 version was employed for the final database, calculations and statistical analysis. The comparison between groups was based on rates. When comparing the percentage of children born each month of the year, birth data were adjusted for 31-day months and leap years. Descriptive statistics and the t-test were employed for the comparisons. Significance was set to 5%. The t-test was carried out two-sided. Data from the medical birth registries were available on the Web. We imported only aggregated data and consequently no Norwegian Data Inspectorate (NDI), Norwegian Social Science Data Services (NSD) or Regional Committees for Medical and Health Research Ethics (REK) approval was necessary.

## 3. Results

During study period the number of births increased by 25% in Sweden and 3% in Norway. Parallelly, the Danish figure was reduced by 14%. During the last three years (2010-2012), there was a drop of 2.7% and 2.1% of the number of births in Denmark and Sweden, respectively. The corresponding figure in Norway was stable. Details are visualized in Figure 1.

**Figure 2:**
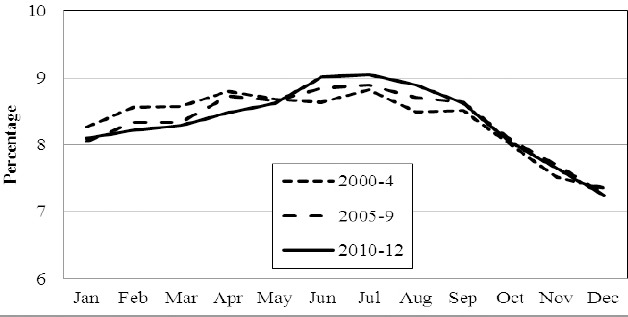
The percentage born in Norway each month of the year in 2000-4, 2005-9 and 2010-12, respectively

**Figure 3:**
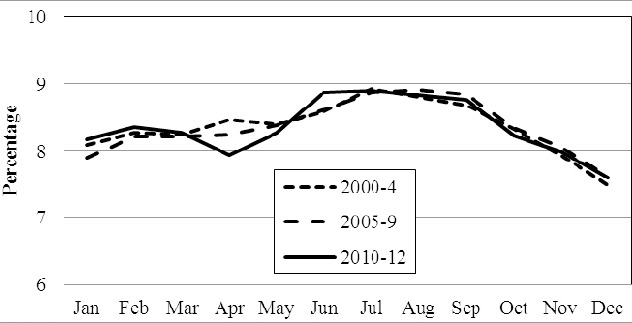
The percentage born in Denmark each month of the year in 2000-4, 2005-9 and 2010-12, respectively

**Figure 4:**
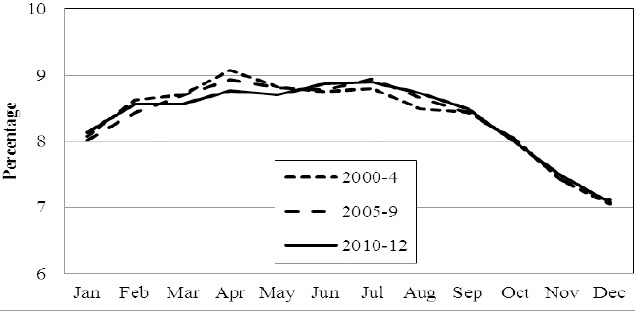
The percentage born in Sweden each month of the year in 2000-4, 2005-9 and 2010-12, respectively

Comparing seasons, most births occurred during summer time (May-August, 35.3%). The individual country figures were: Norway 35.3%, Sweden 35.4% and Denmark 35.1%. The single highest monthly delivery occurred in July and the single lowest in December. This, by implication infers the highest monthly conception to be in October and the lowest monthly conception to be in March. Details are illustrated in Figure 2, 3 and 4.

Whereas we hypothesized a drop in the number of births in Norway within the last quarter of the year, this was not revealed. Such a drop was however observed between January and April (2000-2009 vs. 2010-12, P < 0.04) and parallelly there was a significant increase (P < 0.01) in the share of births during summer time. Details are shown in Figure 2. These findings were not observed in Denmark (figure 3) (P = 0.76) or Sweden (Figure 4) (P = 0.48).

This strongly indicated that Norwegian families had not adapted their family planning to the new national kindergarten reform by changing their birth pattern by fewer births between September and December. Most Scandinavian children were born during summer time. Hospital administrators and owners should be aware of this trend and implement this knowledge when they analyze, plan and organize future summer holidays.

## 4. Discussion

In this study, we have documented that most Scandinavian children were born during summer time (June-September). The clustering of births during summer time may be challenging to health care administrators planning and organizing the obstetric health care. The political reform (Norwegian kindergarten reform) did not influence on time of birth. Other factors seem to be of importance. The single highest monthly delivery occurred in the month of July and the single lowest in December. This, by implication infers the highest monthly conception to be in October and the lowest monthly conception to be in March. These findings differ from many reports in different parts of the world.

### 4.1 Causes of Seasonality of Births

Among the factors reported influencing on birth pattern is the place of living. In example, so-called European and American patterns have been mentioned. In the United States birth seasonality has been reflected in an August/September peak and an April/May one, trough with variation between states in amplitude (Martin et al., 2002; Tita et al., 2001). The nadir has been observed in February. Warren and colleagues (1980) did also report a significant bimodal seasonal trend in the estimated monthly number of conceptions with a major peak from October through January, a minor peak from April to May, and a major trough from June through August. An Indian study (Yadava, 1979) reported their maximum indices of deliveries in the months of August to October and lowest from April to June. In Africa, a study from Mali (Philibert et al., 2013) showed that rates of health-facility-attended deliveries were high in April-June, then fell rapidly and rose again in August-October.

The European pattern has generally been described with a spring peak, a local September peak, and a trough during late fall and early winter. However, there are exceptions. In Croatia, Polasek and colleagues (2005) revealed the highest birth proportion during July-September period, with peak date moving towards the end of summer. A prior Norwegian study (Odegard, 1977) reported that the monthly number of births had a maximum in January-May and a minimum in October-December. The secondary birth maximum was located in September. Similarly, a Finish study (Eriksson et al., 2008) from the Aland Islands did also conclude a general pattern of two peaks, one in March-April and one in September-October.

Seasonality has been related to temperature data. In an American study (Lam et al., 1996), regressions of monthly births on a flexible specification of lagged monthly temperature showed that temperature had quantitatively important effects on both seasonal and non-seasonal variation in births. Summer temperature extremes reduced conceptions in the southern United States, explaining a substantial part of the observed seasonal birth pattern. However, extreme cold showed no evidence of affecting conceptions. The results also showed significant seasonality in births even after accounting for temperature. They concluded that controls for monthly temperature did not explain the spring peak in births in northern Europe. The temperature explanation for variations was difficult, particularly when the birth seasonality between Sweden and the US was different and the seasonal temperature patterns were the same.

Altitude level has also been discussed as a possible explanation. A study from Argentina (Pacual et al., 2002) analyzed the monthly distribution pattern of birth rates in populations located at different geographic altitude levels. Statistically significant seasonal patterns were found in the four geographic regions. The greatest Henry coefficients were observed in spring and summer in the higher regions, whereas they were seen in autumn and winter in the lowlands. It was suggested that these patterns reflected the influence of environmental and socioeconomic altitude-related factors and inter-regional cultural diversity, rather than the influence of geographical altitude per se.

Based on our Scandinavian figures, the previously reported spring peak had been “moved” to summertime. A similar change has been observed in Holland (Haandrikman & van Wissen, 2008). However, in the Dutch study a subsequently change to August/September was revealed, thereby shifting from the European to the American pattern. The overall seasonality pattern was determined by first births (Haandrikman & van Wissen). Consequently, birth seasonality varied by maternal age. The findings stimulated the discussion on the role of planning as a possible cause of birth seasonality. In our study, we expected that planning would reduce the late autumn levels significantly due to the 2009 kindergarten reform. However, this was not revealed. We therefore believe planning was of less importance, at least in Norway.

### 4.2 Summer Peak of Births Has to Be Managed

The Scandinavian birth rate peak during summer time is challenging to health care administrators. Most employees’ wish and have the right to enjoy most of their holiday during summer time. The low birth rate period, especially in November and December, in all Scandinavian countries should be noticed by health care administrators. To optimize the management of obstetric clinics in Scandinavia, courses, educational activities and efforts to upgrade staff´s skills should be located in late autumn and early winter times. When possible health care personnel´s wishes for time off in lieu, holiday and leaves of various kinds should be allocated to these months to lower the pressure on the staff during summer time. Furthermore, incentives in terms of economic resources and/or vacation benefits could be considered to strengthen such a trend.

With today´s contraceptives, time of birth may be planned. Childbirth during summer may be beneficial as this is, in terms of Scandinavian climate, the best time of the year. Children and adults enjoy most of their holidays during this time period. Women´s rehabilitations period after childbirth may be more beneficial during summer. It may also be less challenging to mothers to bring the newborns with them in outdoor settings during this time of the year. Consequently, it could be speculated that more women plan childbirth during summer time. However, this should be further elucidated in future studies.

## 5. Conclusion

The known spring peak in northern Europe has disappeared. The new birth peak is now between June and September. Scandinavian health care administrators should be aware of the new fluctuations in birth rate when they plan hospitals’ annual cycle of activity and manning. The Norwegian kindergarten reform did not influence on the birth rate in the last quarter of the year.
